# Assessing stress using repeated saliva concentration of steroid hormones in dementia care dyads: results from a controlled pilot care music intervention

**DOI:** 10.48101/ujms.v128.9340

**Published:** 2023-05-05

**Authors:** Azita Emami, Töres Theorell, Hyejin Kim, Lars Berglund, Helena Hallinder, Gabriella Engström

**Affiliations:** ^a^The University of Washington, School of Nursing, Seattle, WA, USA;; ^b^Department of Neurobiology, Care Sciences and Society, Division of Occupational Therapy, Karolinska Institutet, Stockholm, Sweden;; ^c^Division of International Public Health, Karolinska Institutet, Stockholm, Sweden;; ^d^Stress Research Institute, Department of Psychology, Stockholm University, Stockholm;; ^e^Department of Adult Health and Gerontological Nursing, Rush University College of Nursing, Chicago, IL, USA;; ^f^Dalarna University School of Health and Welfare, Falun, Sweden

**Keywords:** Caregivers, dementia, music, saliva, stress, cortisol, DHEA-S

## Abstract

**Background:**

Stress-related biomarkers have the potential to provide objective measures of whether interventions directed at people with dementia (PWD) and their family caregivers (FCG) are successful. The use of such biomarkers has been limited by logistical barriers to sample collection.

**Objective:**

Explore saliva concentration of steroid hormones in dementia care dyads during a music intervention.

**Methods:**

Consecutive PWD attending a memory evaluation center and their FCG were allocated to either an intervention-with-music or a non-intervention control group. All were living at home. Stress biomarkers, salivary cortisol and dehydroepiandrosterone sulfate (DHEA-S) samples were collected by the PWD and their FCG, in the morning and evening, 5 days a week, for 8 consecutive weeks. Biomarker concentrations of the intervention and the control groups were compared at week 8, in an intention-to-treat approach with adjustment for baseline value.

**Results:**

Twenty-four PWD in the intervention group and 10 in the control group, and their FCG were included in the analyses. The mean number of morning saliva collections was similar in the intervention and the control groups, ranging from 4.3 to 4.9 per participant weekly during the first 7 weeks, declining to 3.3 during week 8. Median log morning cortisol (pg/mL) among caregivers was lower in the intervention group than in the control group (8.09 vs. 8.57, *P* = 0.0133).

**Conclusion:**

This study demonstrates that music intervention was associated with lower morning saliva cortisol concentrations for FCGs.

## Introduction

As the global population ages, the number of people living with dementia (PWD) is increasing, making the disease a major public health issue. In the early to middle stages of the disease there is a prevailing preference among PWD and their family caregivers (FCGs) for in-home care, which results in increased stress for these dyads (1–3). Unmet physical and emotional needs (e.g. activities of daily living, pain or discomfort, negative emotions) are causes of high stress among PWD, which can lead to a deterioration in the behavioral and psychological symptoms of dementia (BPSD). For caregivers, studies have consistently shown high levels of biological stress levels (4, 5).

Music has been extensively reported as an effective, low-cost intervention that benefits both PWD and their caregivers by helping diminish BPSD at high stress times such as personal care and feeding encounters. However, most studies relied on FCG’s self-report rather than an objective assessment. Recently, attention has focused on developing objective measures that use stress biomarkers. Research shows that active individualized music listening as part of stroke rehabilitation accelerated the healing process of the brain (6).

Methodologically, the invasive nature of the blood collection traditionally required for stress biomarker analysis has been a barrier. In addition to being invasive and uncomfortable, blood collection is expensive, requires a trained phlebotomist, presents infection risk, and is impractical for high-volume, daily, serial collections at home. This led to an interest in saliva collection techniques for assessing cortisol and dehydroepiandrosterone sulfate (DHEA-S) (7, 8). At-home saliva collection has the potential to be an objective, easily implemented, non-invasive way of measuring changes in stress levels that reflect the impact of music or other intervention.

### Stress biomarker daily cycles

Stress triggers the adrenal glands to release cortisol, together with other hormones, in order to maintain homeostasis as summarized by Kristenson et al. (9) Cortisol follows a diurnal rhythm. The levels of cortisol surge immediately after awakening and continue rising for 30 to 45 min. Cortisol then declines rapidly and steadily decreases throughout the day until reaching its nadir around bedtime.

Recent research shows that DHEA-S is correlated with stress levels and the body’s ability to counter adverse effects of stress, which is defined as long-lasting energy-mobilization without effective recuperation (10). A greater ratio of cortisol-to-DHEA-S during acute stress responses among persons with higher long-lasting stress levels than among those with lower levels (11) makes salivary DHEA-S a relevant stress biomarker in this study. Such a ratio indicates an adverse balance between energy mobilization and regeneration that may be particularly harmful in caregivers for persons with dementia since many acute stress situations can be expected to arise in their daily routine (10). Ouanes et al. (12) have shown that high cortisol levels and a high cortisol/DHEA-S ratio in cerebrospinal fluid are associated with cognitive decline.

During interventions aimed at reducing stress levels and increasing capacity to handle stress, a diminished cortisol-to-DHEA-S ratio is a favorable development. Most studies to date using salivary biomarkers of stress have made only single-point measures before and after an extended period of intervention, rather than the longitudinal daily evaluation of salivary biomarkers that would more accurately capture fine-grained changes in response to music or other intervention programs (7, 13–15). In this study, we aim to explore saliva concentration of steroid hormones in dementia care dyads during a music intervention. A prior preliminary proof-of-concept study examined only an intervention group (16).

## Methods

### Sample and setting

**Figure 1:**
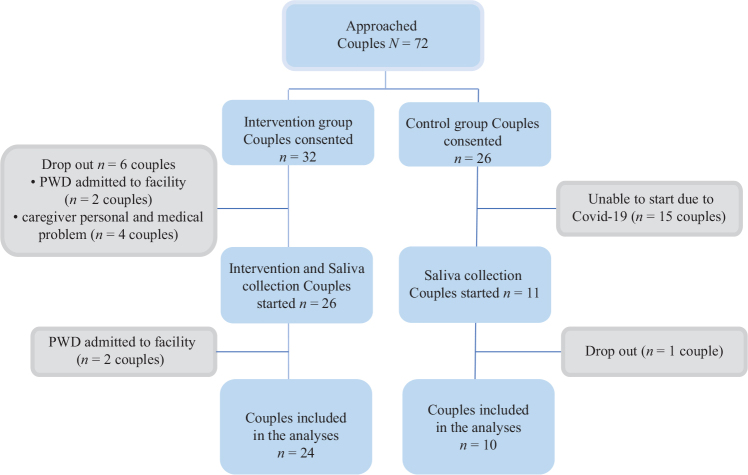
Participant flow diagram.

We used a two-group, non-randomized open trial to explore the effects of music on physiological markers of stress, cortisol and DHEA-S in saliva, among PWD and their FCG. PWD and FCG, who visited a memory care center, were approached by a study coordinator via phone. We collected the data between November 2018 and March 2020. Eligible PWD and their FCG were allocated as dyads in a 1:1 ratio to a music-based intervention group or comparison group (Figure 1). A total of 72 dyads (PWD and FCG) were approached. Of those 72, 18 dyads declined to participate and the remaining 58 dyads (32 for intervention group and 26 for comparison group) consented to participate in this study. A total of 34 dyads (24 in the intervention group and 10 in the comparison group) were included in the analysis. We included PWDs who 1) were diagnosed with dementia; 2) were in early to severe stage of dementia as defined by a Global Deterioration Scale (GDS) score of 4 or greater (17); 3) lived with a FCG; and 4) provided written informed consent (or proxy consent from the caregiver) to participate. FCG participants were included if they 1) were at least 18 years of age and 2) provided written informed consent to participate. We excluded PWD and FCG who have active mental disorders (e.g. severe depression and anxiety disorder) or those who previously participated in other music therapies or music interventions.

### Saliva collection and intervention education

After obtaining an informed consent form, both the intervention and the control group dyads received an education session on saliva collection from the study coordinator. The saliva collection has been described elsewhere (16, 18, 19). Briefly, the education session provided written instructions on each step of saliva collection and hands-on training. Participants were instructed to collect saliva over an 8-week period. Participants collected their saliva in the morning and in the evening for 5 days, from Sunday evening to Friday morning. Participants were allowed to collect the samples based on their own diurnal cycle rather than at specific times.

Collecting saliva is a simple and non-invasive procedure that does not require much equipment or technical knowhow. This allows for multiple samples to be taken from the same individual over time. The cortisol levels in a person with normal cortisol regulation capacity upon waking provide an indication of the expected stress level during the day. Therefore, the difference between cortisol levels in the morning and evening can serve as a proxy measure of the awakening response.

A trained research assistant who completed the dementia-specific online education session (e.g. how to communicate with PWD) picked up the saliva every morning at participants’ homes and delivered the samples to the Biobank at the Karolinska Institutet for handling until they were shipped for analyses. With COVID-19 lockdowns and social distancing measures, it became increasingly difficult to pick up the saliva samples at the participants’ homes, leading to cancellation of the study in March 2020. At that time, not all participants had completed the full 8-week saliva collection. The samples were assayed using radioimmunoassay at the Truly Labs, Lund, Sweden.

In addition to the saliva collection session, the intervention group also received an education session about the music-based intervention. The music-intervention has been described elsewhere (16, 18, 19). Briefly, the study coordinator provided information about the music-based intervention (e.g. benefits of music and how to access the intervention). PWD and FCG participated in the online, in-home music intervention whenever they wanted or could attend, together or alone at their convenience, throughout the study. They were encouraged to listen together and to create a daily routine for the music listening.

### Measures

Mean values (picograms per milliliter; pg/mL) of four indices were used for each week of the study period to measure the levels of stress in the body among PWD and FCG. Firstly, cortisol concentrations (i.e. primary indicator of stress) were measured using the morning samples and evening samples. Secondly, DHEA-S concentrations were measured using participants’ morning samples. Thirdly, diurnal cortisol variation was assessed by subtracting evening cortisol concentrations from morning cortisol concentrations. Fourthly, the ratio of cortisol to DHEA-S (i.e. indicator of adverse chronic stress) was measured using participants’ morning saliva samples.

### Statistical analysis

Analyses were performed using SAS^®^ version 9.4 (SAS Institute Inc., Cary, NC, USA). The level of statistical significance for two-tailed hypothesis testing was set at 0.05. No adjustments for multiple tests were made. Firstly, data were screened for anomalies to check for outliers and missing data. Participants who did not complete the questions regarding a given sample were considered to have missing saliva data for that sample. Saliva samples with insufficient saliva for the assay were treated as missing. Due to highly skewed distributions of saliva markers, all values were transformed using natural logarithms. The appropriate descriptive statistics were used based on the levels of measurement and data distribution (means with standard deviations [SD], median with interquartile ranges [IQR], frequency [*n*], percentage [%], and geometric means).

All analyses were made separately for PWD and for their FCGs. The analysis was based on primary endpoints (PEs), which are the mean values of the last available week (i.e. last observation carried forward [LOCF] analysis). Carrying forward an intermediate value is a conservative estimate of outcome trajectory when treated patients are assumed to improve gradually. Since true baseline values (i.e. measurements before intervention) did not exist, mean values from the first week of the study period were used as ‘pseudo’ baseline values. The analysis was based on intention-to-treat with intervention/control group comparisons of PEs at 8 weeks with adjustments for baseline values.

The distribution of residuals in the regression models of PEs on intervention/control group and baseline values, was examined for normality with Shapiro–Wilk’s test, where *w* > 0.95 indicates normal distribution. For normally distributed PEs, linear regression models of PEs on intervention/control group and baseline values were estimated. Means adjusted for baseline values, by intervention/control group, *P*-values for test of the null hypotheses and 95% confidence intervals (CIs) for comparisons of adjusted intervention/control group means were presented. For non-normally distributed PEs, intervention/control group comparisons were performed using Willetts’s residual method (20). The residuals in the linear regression of PEs on baseline values were calculated and compared between intervention and control groups using Wilcoxon two-sample test. The medians were adjusted for baseline values, by intervention/control groups; *P*-values for tests of null hypotheses and 95% Moses CIs for median differences between the intervention and control groups were calculated.

To examine the robustness of the LOCF method we did a sensitivity analysis using multiple imputation with the fully conditional specification method based on the intervention/control group variable and previous values of each PE (50 imputations), for calculation of estimated effects and *P*-values.

Number of weeks elapsed and number of saliva collections per week were compared between the groups with Wilcoxon two-group test.

Due to shorter data collection time for the control group, a sensitivity analysis was performed with week 6 as the last available observation week (i.e. observations from weeks 7 and 8 were disregarded).

## Results

Table 1 reports baseline characteristics of PWD and the FCG. Among the participating dyads who started the saliva collection, 92% (*n* = 24) in the intervention group and 90% (*n* = 10) in the control group were included in the analyses (Figure 1). Median participation time was 8.0 weeks for the intervention group and 6.5 weeks for the control group (*P* = 0.06).

**Table 1:** Baseline sample characteristics for persons with dementia and the family caregivers in the intervention and control group.

Variable	Persons with dementia			Family caregivers		
	All (*N* = 34)	Intervention (*n* = 24)	Control (*n* = 10)	All (*N* = 34)	Intervention (*n* = 24)	Control (*n* = 10)
**Age** M ± SD (range)	―	79 ± 8.5 (60–92)	79 ± 5.0 (72–96)	―	73 ± 11.5 (37–90)	77 ± 6.0 (69–86)
**Sex** *n* (%)						
Female	―	7 (29)	4 (40)	―	16 (67)	6 (60)
Male	―	17 (71)	6 (60)	―	8 (33)	4 (40)
**Activities of daily living**^a^ M ± SD (range)	2.4 ± 1.7 (0–6)	2.4 ± 1.7 (0–6)	2.4 ± 1.7 (0–6)	―	―	―
**Months since dementia diagnosis** (range)^b^	20.2 ± 14.6 (2–60)	20.1 ± 14.0 (5–57)	20.3 ± 16.7 (2–60)	―	―	―
**Global Deterioration Scale**^c^ M ± SD (range)	4.8 ± 1.0 (4–7)	4.9 ± 1.1 (4–7)	4.4 ± 1.0 (4–6)	―	―	―
**GDS**^c^**,** *n* (%)						
Moderate cognitive decline	17 (50)	12 (50)	5 (50)	―	―	―
Moderately severe cognitive decline	8 (24)	4 (17)	4 (40)	―	―	―
Severe cognitive decline	7 (21)	6 (25)	1 (10)	―	―	―
Very severe cognitive decline	2 (6)	2 (8)	0 (0)	―	―	―
**Perceived general health^b^,** *n* (%)						
Excellent	―	―	―	1 (3)	0 (0)	1 (10)
Very good	―	―	―	11 (34)	8 (35)	3 (30)
Good	―	―	―	13 (38)	11 (48)	2 (20)
Fair	―	―	―	8 (24)	4 (17)	4 (40)
Poor	―	―	―	0 (0)	0 (0)	0 (0)

Note: M ± SD = mean ± standard deviation.

GDS: Global Deterioration Scale.

a ADL scores of PWD were reported by their FCG.

b One participant in the intervention group was excluded due to the incompleteness of the item.

c GDS scores of PWD were reported by the study coordinator.

There were no statistically significant differences between the intervention and control groups with regard to timing of saliva collection. The collection of the morning sample occurred between 07.20 and 07.45 in the intervention group FCGs and between 07.32 and 07.42 in the control group FCGs. Corresponding timing of saliva collection for PWD in the intervention group occurred between 07.41 and 08.07 and in the control group between 07.46 and 08.18.

**Figure 2:**
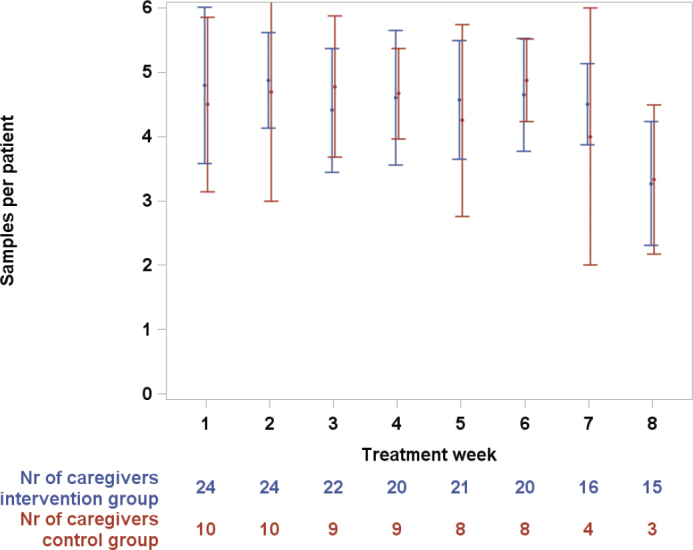
Number of saliva tests per family caregiver by treatment week for intervention and control groups. Mean values with error bars (std) and number of remaining caregivers by treatment week.

Of five potential morning samples for each person per week, the mean number of saliva collections per FCG was similar between the intervention and control groups, ranging from 4.3 to 4.9 per week during the first 7 weeks and declined to 3.3 during week 8 (see Figure 2). Results for PWD were similar.

### Stress biomarkers in dementia care dyads

Table 2 shows medians with IQRs for log morning cortisol in the PWD and FCG intervention and control groups. The baseline values are shown at first and then the corresponding values at follow-up, with adjustment for baseline (making intervention and control group comparable) and LOCF. Then the adjusted group difference at follow-up is presented as 95% confidence limits and *P*-value for the group difference. In the PWD group, the log morning cortisol minus evening cortisol results as well as the log DHEA-s and the log cortisol/DHEA-s results are presented with medians as well. In the remaining analyses means with SDs are presented. The reason for choice of median versus mean is that median was used when a variable’s distribution differed from normality and vice versa.

**Table 2:** Saliva concentrations of steroid hormones at baseline and LOCF^1^ for persons with dementia and family caregivers in intervention and control groups.

Variable		Group	Baseline mean (std)/ median (interquartile range)	LOCF^1^ mean (std)/ median (interquartile range)	Adjusted LOCF^1^ mean/ median	Adjusted group difference at follow-up		
						*P*	Lower 95% CL	Upper 95% CL
**Persons with Dementia**	**Log morning cortisol (pg/mL)**	Intervention *n* = 24	8.05 (5.96–8.34)	7.85 (6.03–8.35)	8.04			
		Control *n* = 10	8.62 (8.03–9.29)	8.58 (8.19–8.88)	8.10*	0.38	−0.644	0.169
	**Log morning DHEA-s (pg/mL)**	Intervention *n* = 24	7.95 (0.91)	7.89 (1.01)	8.18			
		Control *n* = 10	8.90 (0.68)	8.79 (0.97)	8.16**	0.94	−0.506	0.545
	**Log morning cortisol/morning DHEA-s**	Intervention *n* = 24	−0.60 (1.43)	−0.62 (1.54)	−0.51			
		Control *n* = 10	−0.33 (1.13)	−0.13 (1.05)	−0.48**	0.90	−0.603	0.535
	**Log morning cortisol (pg/mL) minus log evening cortisol (pg/mL)**	Intervention *n* = 24	1.42 (0.89–1.90)	1.36 (0.66–1.84)	1.42			
		Control *n* = 10	1.18 (0.42–1.62)	1.01 (0.37–1.66)	1.73*	0.45	−0.555	0.635
	**Log evening cortisol (pg/mL)**	Intervention *n* = 24	6.10 (1.48)	6.28 (1.71)	6.76			
		Control *n* = 10	7.84 (1.64)	8.14 (1.56)	6.98**	0.55	−0.977	0.529
**Family Caregivers**	**Log morning cortisol (pg/mL)**	Intervention *n* = 24	8.07 (6.25–8.55)	8.06 (6.05–8.35)	8.09			
		Control *n* = 10	8.22 (7.91–8.50)	8.60 (8.37–8.76)	8.57*	0.013	−0.910	−0.124
	**Log morning DHEA-s (pg/mL)**	Intervention *n* = 24	8.30 (7.38–8.66)	7.78 (7.14–8.54)	8.30			
		Control *n* = 10	7.78 (6.84–8.55)	7.72 (7.50–8.51)	8.51*	0.44	−0.947	0.506
	**Log morning cortisol/morning DHEA-s**	Intervention *n* = 24	−0.12 (−1.62 to 0.34)	−0.34 (−0.97 to 0.43)	−0.42			
		Control *n* = 10	0.51 (−0.20 to 1.25)	0.93 (0.20–1.10)	0.28*	0.36	−1.135	0.454
	**Log morning cortisol (pg/mL) minus log evening cortisol (pg/mL)**	Intervention *n* = 24	1.62 (0.80)	1.59 (0.86)	1.51			
		Control *n* = 10	1.19 (1.00)	1.47 (0.75)	1.64**	0.64	−0.683	0.425
	**Log evening cortisol (pg/mL)**	Intervention *n* = 24	6.28 (4.52–6.95)	6.18 (4.63–7.34)	6.45			
		Control *n* = 10	6.73 (6.44–7.09)	6.72 (6.50–7.06)	6.74*	0.86	−0.657	0.476

1 Last observation carried forward,

* Non-normal distribution, WRM = Willett’s residual method/median,

** Normal distribution, LR = Linear regression/mean.

LOCF: last observation carried forward; CL: Confidence limit ; DHEA-S: dehydroepiandrosterone sulfate.

**Figure 3:**
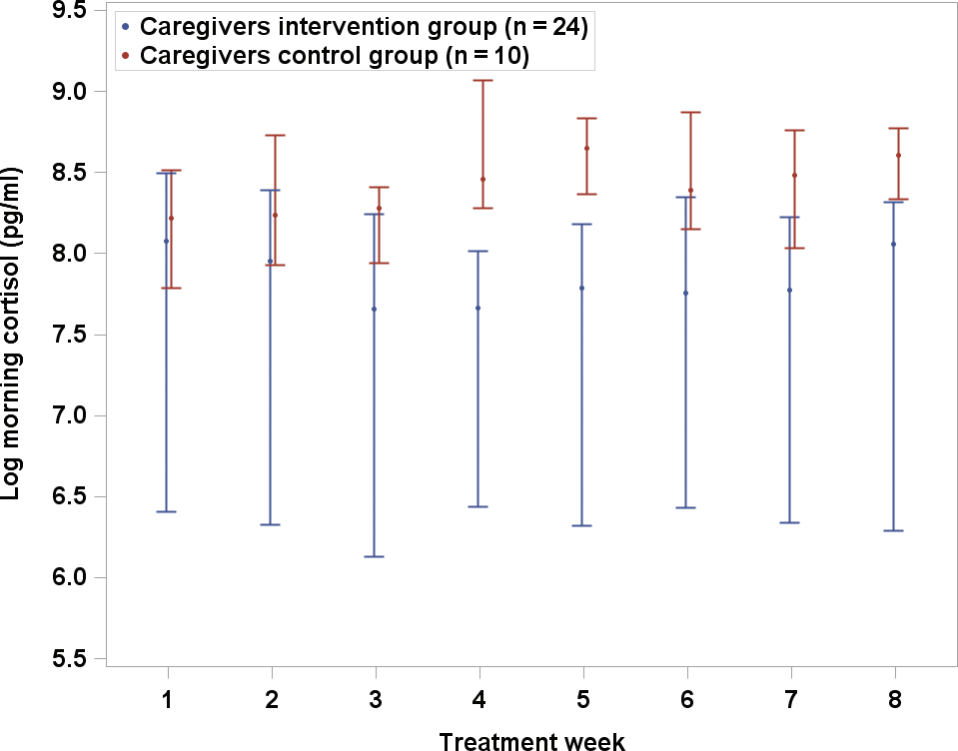
Log morning cortisol by treatment week for family caregivers in intervention and control groups. LOCF median values with error bars (95% confidence interval).

**Figure 4:**
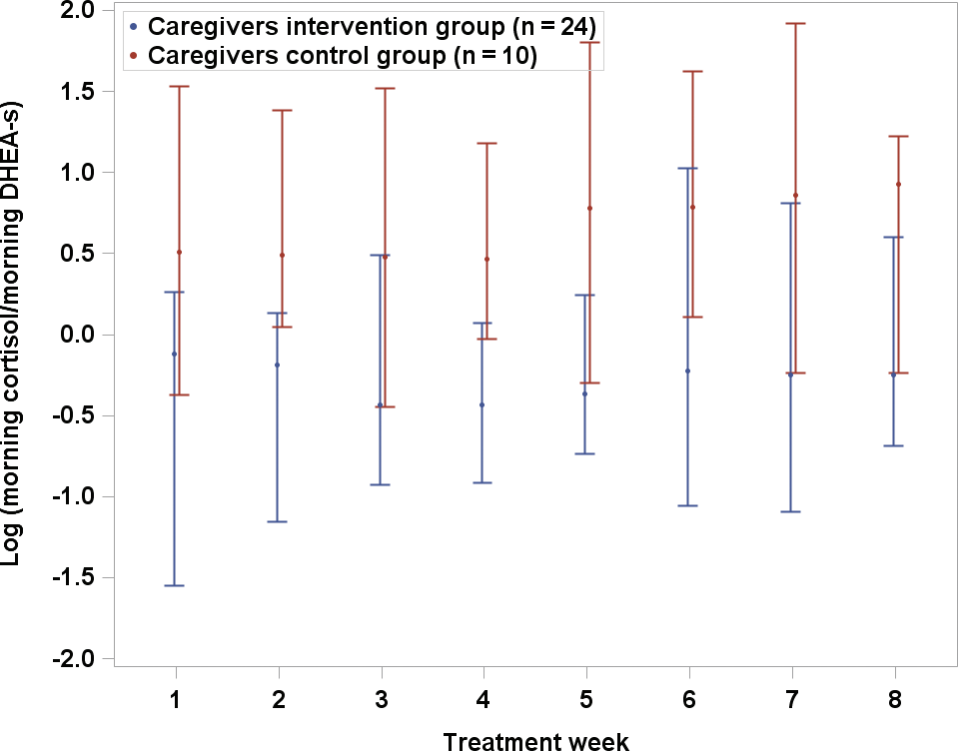
Log morning cortisol/DHEA-s by treatment week for family caregivers in intervention and control groups. LOCF median values with error bars (95% confidence interval).

Although we did not find significant differences for PWD (Table 2), FCG in the intervention group showed lower adjusted log morning cortisol LOCF than the control group (*P* = 0.013, Figure 3). Adjusted medians for intervention and control groups were 8.09 and 8.57 log pg/mL, respectively. A total of 95% CIs for difference between adjusted medians was −0.91 to −0.12. No other adjusted LOCF differences between groups were statistically significant: The corresponding non-significant results for log morning cortisol/DHEA-s are shown in Figure 4.

In the sensitivity analysis with week 6 as the last available observation week, results were similar. FCG in the intervention group showed lower adjusted log morning cortisol LOCF than the control group (*P* = 0.047) in this sensitivity analysis and adjusted medians for intervention and control groups were 7.54 and 8.02 (log pg/mL), respectively. When we used multiple imputation with 50 imputations for estimated effects and *P*-values the results were similar to the LOCF analysis. For example, for morning cortisol among FCG the *P*-value was 0.0205 and the difference between groups were comparable to LOCF results.

## Discussion

### Findings for the family caregivers

FCGs participating in the music intervention had lower saliva morning cortisol concentrations than the control group in the final week. The geometric mean at the final week was 2,039 pg/mL in the intervention group, and 3,463 pg/mL in the control group, adjusted for baseline value level.

Kristenson et al. (9) emphasize that chronic stress is associated with disturbed regulation of cortisol excretion, often resulting in a ‘flattened’ circadian cortisol rhythm with relatively low morning and high evening cortisol. Previous work (16), based on only a music intervention group, showed a statistically significant difference between morning and evening cortisol levels among the caregivers. There was little evidence of disturbed regulation of saliva cortisol due to chronic stress despite the well-known burden of providing at-home care for a person with dementia. Thus, the participants, in particular the caregivers, should be regarded as subjects with retained ability to regulate cortisol levels. What we could hypothesize in this study is a lowered morning cortisol as well as a lowered evening cortisol and a lowered cortisol/DHEA-s level with a lowered stress level.

Prior work (16) based on the intervention group in this study showed pronounced day-to-day variations. This is an important factor that should be considered in all such studies, because these variations complicate the analysis of whether there are significant change patterns. In the present study this was handled by averaging all data collected during a week.

An extra sample was collected in the morning, half an hour after awakening. In three cases when the first morning sample failed, this second sample was used for analysis. The most common pattern in healthy individuals is that there is a rise in cortisol concentration between awakening and half an hour later. We decided not to analyze this ‘morning rise’ (much smaller than the normal morning-evening difference), which is a recommended stress measure, since it was impossible to follow the strict rules surrounding this kind of assessment (21).

The most likely explanation for the cortisol level findings in the caregiver group is that there was a lower morning stress level in the caregivers participating during the 8 weeks of intervention with music at times of personal care than in the corresponding control group. The morning routines may be particularly stressful for the caregivers, which could be the reason why the findings are more pronounced in the morning than in the evening. In the previous report (16) there is anecdotal evidence describing decreased within-dyad conflicts as a consequence of the music intervention. As reported in a separate article (19) caregiver ratings of stress and depression improved more in the intervention than in the control group.

There were pronounced differences between individual couples in the intervention group, as reported previously (16). Our individual analyses indicated that approximately one-fourth of the dyads may have markedly benefited from the music intervention (16).

Brown et al. (5) published the results of a randomized evaluation of the effects for FCG of a mindfulness training program that was compared with a program with improved social support. The programs lasted for 2 months and saliva cortisol was assessed six time points during one day, at each of the 3 study phases (pre- and post-intervention, and 3-month follow-up. No differences were found between the intervention groups. Whether our positive findings for the caregiver group with regard to morning cortisol are due to the interventions or differences in the schedules for saliva cortisol collection is not known.

We hypothesized that the ratio between cortisol and DHEA-s would improve due to the intervention with music during periods of personal care. The comparison with the control group did not confirm this. However, as reported (16), one-fourth of the caregivers in the intervention with music group showed such improvement, although the difference was not statistically significant (*P* = 0.36). This needs to be examined in more depth in the future.

### Findings in the persons with dementia

There were no statistically significant differences in any of the study variables in the PWD group. For morning cortisol there was greater decrease (improvement) in the intervention group than in the control group, but the difference in improvement was not statistically significant (*P* = 0.38). One reason for the lack of statistically significant group differences for the PWD group may be that there was rather rapid disease progression in some of the people. These PWDs are also vulnerable to other kinds of illnesses, for example massive infections. Such episodes represent stressful events that cause hormonal levels (16) to fluctuate.

### Study intention and limitations

The present study had a number of limitations that should be considered when assessing the outcomes.

- The COVID-19 pandemic resulted in a reduced size and collection duration for the control group, complicating and compromising the attempt to establish statistical significance of the intervention effects.
- Cohort sizes were small.
- The study populations were non-randomized. However, the PWD groups in the intervention and control conditions were comparable in age, Activities of daily living (ADL) functional level, and months since diagnosis.
- The median period of data collection for the control group was shorter than for intervention group (8.0 and 6.5 weeks, respectively). However, the significance of the intervention/control group difference in the caregivers was retained when the comparison period was limited to the initial 6 weeks.
- Data from the intervention group were not collected during the same year as those from the control group. The saliva concentration of cortisol, particularly in the morning, may show seasonal variations. Persson et al. (22) studied saliva cortisol measures in 24 working men and women over the course of a year. The differences in morning saliva cortisol were pronounced and statistically significant between seasons. Since all the saliva sampling in the present study took place from January to April, when the differences are not so pronounced, this is unlikely to have caused significant bias.
- We did not gather data on the timing or frequency with which the FCGs chose to utilize a music intervention, so it is impossible to determine if personal care encounters increased with rising levels of dementia and if music was deployed more or less frequently based on an increase in such encounters.

## Conclusion

We have previously shown that serial, in-home saliva collection on a daily basis by study participants over a multi-week period is feasible (18). In this study we demonstrate that music intervention was associated with lower morning saliva cortisol concentrations for FCGs.

A strength of this study is that cortisol and DHEA-S were measured 5 days per week throughout an 8-week period, offering insight into participants’ diurnal cortisol and DHEA-S levels in general. This process enabled collection of a large number of observations for each participant, and established the potential for using this methodology to objectively measure responses in stress levels to music and other interventions directed at modifying the adverse BPSD.

Having such an objective yardstick is important, because progressively worse BPSDs are a frequent reason for PWDs being institutionalized. Developing successful interventions requires a reliable, repeatable means of measuring the outcomes of such interventions for both PWDs and their caregivers. Evidence suggests that music, by stimulating the neural pathways in the brain, affects physiological responses and mediates and moderates behavior in PWD (23, 24).

We have demonstrated a viable alternative to the use of self-report questionnaires for capturing the impact of a music intervention on caregivers and particularly on PWD. As the latter become progressively less able to self-report, data become skewed to increasingly reflect the perceptions of the caregiver rather than the physiological response of the PWD. This adds urgency and importance to the need for developing objective measures of stress. In a forthcoming paper we will examine day-to-day variability of salivary cortisol and DHEA-S levels both among PWD and FCGs and evaluate how a music intervention for stress affects variability in PWD and FCGs. Furthermore, to facilitate design of future PWD and FCG dyad studies we will present how many sample days one needs for establishing salivary cortisol and DHEA-S differences between intervention and control groups for a given number of dyads.

A more definitive result will require a larger Randomized Control Trial (RCT) that controls for confounding variables such as seasonality, as well as acute infections and other temporary stressors such as changes in care personnel, family conflicts, and medical encounters.
